# Changes in grocery shopping behaviour among low-income households during the COVID-19 pandemic

**DOI:** 10.1017/S1368980024002672

**Published:** 2025-01-07

**Authors:** Angela CB Trude, Caitlin M Lowery, Gabriela M Vedovato, Shahmir H Ali, Josephine M Dudzik

**Affiliations:** 1 Department of Nutrition and Food Studies, Steinhardt School of Culture, Education, and Human Development, New York University, 411 Lafayette St, 5th floor, New York 10003, NY, USA; 2 Department of Nutrition, Gillings School of Public Health, University of North Carolina at Chapel Hill, 135 Dauer Dr, Chapel Hill 27599, NC, USA; 3 Institute of Health and Society, Federal University of Sao Paulo, 136 Silva Jardim, Santos 11015-020, SP, Brazil; 4 School of Global Public Health, New York University, 708 Broadway, New York 10003, NY, USA

**Keywords:** Food security, Food assistance, Diffusion of innovation, Nutrition policy

## Abstract

**Objective:**

The Supplemental Nutrition Assistance Program (SNAP) Online Purchasing Pilot (OPP) authorised the use of SNAP benefits online in Maryland in May 2020. We assessed shopping behaviour and intentions associated with uptake and intended future use of online grocery shopping during and after COVID-19 among SNAP-eligible households.

**Design:**

In this mixed-methods study, participants completed a survey on online grocery shopping, and a purposefully sampled subset participated in focus groups or in-depth interviews between November 2020 and March 2021.

**Setting:**

Predominantly urban households in Maryland

**Participants:**

Primary shoppers of SNAP-eligible households with young children (*n* 310)

**Results:**

Most participants reported first shopping for groceries online after the OPP was implemented (57 %). Families who purchased groceries in-store less frequently were less likely to report ever buying groceries online (rate ratio (RR): 0·66, 95 % CI 0·46, 0·93) compared with weekly grocery shoppers. Shoppers who intended to purchase more groceries online in the next 6 months were more likely to have online shopping experience, although this differed by timing of online grocery service adoption. Participants reported more negative attitudes towards in-store grocery shopping during the pandemic than prior to its onset and cited COVID-19 as a motivator for ordering groceries online in focus groups. Most participants who had shopped online planned to continue after the pandemic (79 %).

**Conclusions:**

Most participants who shopped online started during the COVID-19 pandemic and considered the pandemic a key motivator. Findings suggest that low-income households will continue to shop online, affirming the need for policies that promote equitable access to healthy food online.

Online grocery services are an emerging component of the food system that have the potential to increase access to healthy food, particularly for communities with few supermarkets and limited access to transportation^(1,2)^. In July 2020, nearly 40 % of respondents in a nationally representative survey reported having shopped for groceries online (ever)^(3)^. The COVID-19 pandemic and social distancing policies sparked a remarkable increase in online grocery shopping use^(4)^, precipitated by stay-at-home orders and health and safety concerns related to the pandemic^(5,6)^. In fact, online grocery shopping sales have increased from $4 billion in March 2020 to $7·1 billion in March 2021 in the USA^(7)^. However, barriers to the adoption of online grocery services have been identified, particularly among older, rural and/or underserved populations, and participants of federal food assistance programmes, such as the Supplemental Nutrition Assistance Program (SNAP) and the Special Supplemental Nutrition Program for Women, Infants, and Children (WIC)^(1,8–12)^.

Prior to the US Department of Agriculture’s (USDA) rolling implementation of the SNAP Online Purchasing Pilot (OPP) in March 2020, SNAP participants were not able to use their benefits to purchase groceries online^(13)^. The OPP was initially implemented in eight states, including Maryland, and rapidly expanded across the USA in response to COVID-19^(13)^. Only 3·3 % of SNAP participants used their benefits online from June to August 2020 in Maryland, although among those who did, the average transaction size was equivalent to 21 % of the maximum benefit^(14)^. Online grocery uptake was reported to be even higher in another national study among adults receiving SNAP after the SNAP OPP expansion, where 54 % reported online grocery shopping between October 2020 and November 2021, with higher odds of uptake for those between 18 and 44 years old than older adults, for households with children, or food-insecure^(15)^.

The limited initial use of SNAP benefits online aligns with findings from previous studies, which found mixed attitudes towards online grocery shopping among individuals participating in federal food assistance programmes^(8–10)^. Prior research has identified concerns regarding cost, product quality, and delivery wait times as deterrents of online grocery shopping^(8)^. On the other hand, ordering groceries online was perceived as less stressful and resulted in fewer self-reported impulse buys than in-store purchases among women enrolled in WIC^(10)^, to reduce food insufficiency among families of low income^(16)^. The additional benefits of online grocery services identified by low-income households included saving time, delivery of heavy or bulky items^(17)^, and the ability to use SNAP benefits online in some pilot studies^(9,18)^.

We hypothesised that the COVID-19 pandemic was a situational factor that motivated individuals to start buying groceries online. Prior research indicates situational factors, such as health problems or having a baby, may play a role as triggers for the uptake of online grocery shopping^(19)^. However, the factors leading to the adoption of online grocery shopping and those associated with continued use of the service may differ. Literature shows divergence in relation to the discontinuation of online grocery shopping when the initial trigger, that is, the situational factor, disappears^(19,20)^. Despite the abundance of studies on online grocery shopping during the pandemic, it is unclear whether the use of online grocery shopping will continue after the pandemic. Thus, it is necessary to better understand the attitudinal, behavioural and contextual factors related to the adoption, use and continuation of online grocery services, particularly among low-income households.

The primary objective of this study was to identify purchasing behaviours and intentions associated with adoption of online grocery services, using a mixed-methods approach. Second, we explored changes in attitudes, social norms and behaviour related to in-store grocery shopping during the COVID-19 pandemic. Finally, we assessed the association between attitudinal factors and intent to continue or increase shopping for groceries online in the future.

## Methods

### Study design

The data analysed in this study were collected from November 2020 to March 2021 as part of a larger mixed-methods study of online grocery shopping behaviours among SNAP-eligible households with young children living in Maryland, which has been described elsewhere^(12)^. The researchers primarily utilised online recruitment methods, including social media ads, ResearchMatch.org and school- and community-based listservs, and invited patients from a paediatric nutrition clinic for underserved families to take part in an online survey. All research recruitment materials and data collection instruments were only available in English; thus, ability to read and communicate in English was an initial eligibility criterion. The study provided interested individuals with a unique link to a Qualtrics eligibility screener: (i) adults living in Maryland with a child aged ≤ 8 years, (ii) who self-identified as the primary shopper for their household (i.e. purchasing groceries at least once/month) and (iii) reported household income ≤ 130 % of the federal poverty level and/or participation in SNAP in the previous 12 months were eligible to take the online survey on grocery shopping habits.

### Sample

A total of 310 individuals completed the survey and were included in the quantitative analytic sample. Using purposeful sampling, a subset of survey participants (*n* 214) was invited to complete a screener assessing interest in participation in an online focus group. Ninety-five individuals who completed the pre-focus group screener expressed interest in participating and were invited to attend a focus group. We used stratified sampling to ensure that the qualitative data included the perspectives of SNAP and non-SNAP participants, and those with and without prior experience online grocery shopping.

Five to ten individuals were invited to attend each focus group discussion, although mean attendance ranged from 3 to 4 individuals per focus group (*n* 11 groups). If fewer than three individuals attended a focus group, the facilitators conducted individual in-depth interviews (*n* 5). Forty-four participants attended a focus group or in-depth interview, but two were excluded from the analysis as they lived with another participant who reported shopping for groceries more frequently and were thus ineligible for the study, for a final qualitative sample of forty-two participants.

### Data collection

The quantitative survey questions aimed to assess attitudes, norms and behaviours related to online and in-store grocery shopping, according to the domains of the theory of planned behaviour^(21)^. Prior to recruitment, the researchers consulted key stakeholders working in food access among low-income populations using the Delphi method^(22)^ to improve survey questions and conducted eight cognitive interviews to enhance the clarity of the instrument. Survey questions included thirty-four Likert scale questions on attitudinal factors and perceived barriers to in-store and online grocery shopping, as well as sociodemographic characteristics and typical grocery purchasing behaviours.

The qualitative interview guide was also informed by the theory of planned behaviour and preliminary findings from the quantitative survey. The researchers made minor iterative revisions to the interview guide throughout the data collection process to explore insights from prior focus groups. Because of the stratified sampling strategy, the interview guide was modified slightly for different participant groups to ensure questions were relevant (e.g. SNAP participants who have never shopped online and SNAP-eligible non-participants who have shopped online).

All focus group discussions and interviews were conducted in English using Zoom video conferencing, which has been observed to be an effective and valid method in interview-based qualitative research, particularly during the COVID-19 pandemic^(23,24)^. Consent was obtained via a short survey prior to participation, and participants were encouraged, but not required, to turn on their videos to improve engagement in the session. Two research assistants, trained in qualitative research methods at the graduate level, facilitated the sessions. Interview and discussions were transcribed verbatim, but responses were de-identified.

### Data analysis

#### Quantitative data analysis

In the parent mixed-methods study^(12)^, researchers conducted a confirmatory factor analysis to create standardised factor scores to explain individual attitudinal and social determinants of online grocery shopping behaviour. The present analysis builds on the attitudinal and social standardised factor scores, to further examine its association with future intention to increase or continue shopping for groceries online. The confirmatory factor analysis yielded a five-factor solution. Factor 1 was modified to exclude one item, ‘In the next 6 months, I expect to buy groceries online more than I currently do’, as future intention to shop online was an outcome of interest in the present study. After re-running the factor analysis, the five-factor solution was still preferred, with minimal change to the Goodness of Fit statistics: Factor 1 – Facilitators (seven items, e.g. ‘Buying groceries online is helpful to me’), Factor 2 – Fees (two items, e.g. ‘I don’t mind paying for service fees’), Factor 3 – Perceived Control (nine items, e.g. ‘It does not take too long to search for specific products or labels online’), Factor 4 – Access (two items, e.g. ‘I have access to a reliable internet connection to purchase groceries online’) and Factor 5 – Pickup (two items, e.g. ‘Picking-up my groceries in store is more convenient than shopping in-store’). Goodness of Fit statistics for root mean square error of approximation (RMSEA) were 0·075 (90 % CI 0·66, 0·084), and standardised root mean square residual (SRMR) was 0·050. All standardised factors had a Cronbach’s *α* > 0·70 (range 0·73–0·89).

Descriptive analyses were conducted, including frequencies, means and standard deviations using Stata 16.1^(25)^. The primary outcomes of the study were (1) use of online grocery services and (2) early adoption of online grocery shopping. The concept of early adopters, a component of the diffusion of innovation theory, was popularised by Katz et al. in 1963^(26)^ and describes individuals who are among the first to adopt a new trend or behaviour and play a role as opinion leaders in their social circles. Multivariate robust Poisson regression models were used to assess the association between shopping behaviours and intentions and shopping for groceries online (ever), and between shopping behaviours and intentions and early adoption of online grocery services. Robust Poisson regression models allow us to estimate unbiased risk ratios for binary response variables, rather than OR, which are challenging to interpret when the outcome is common. The *t* test statistic was used to compare for changes in means of shopping attitudes before and during the COVID-19 pandemic. Finally, robust Poisson regression models were used to examine the relation between the standardised factors and intent to shop more online in the following 6 months than previously, and for those who had ever shopped online, between the standardised factors and intent to continue shopping for groceries online after the pandemic. As a sensitivity analysis, we also ran logistic regression models in place of the robust Poisson regressions to test the relation between shopping intentions/behaviours and online grocery shopping adoption, and the relationship between attitudes and intention to continue shopping online or shop more online in the future, which yielded similar results (see online supplementary material, Supplemental Table 3).

#### Qualitative data analysis

Qualitative data were transcribed, and an initial codebook was developed from a sample of transcripts informed by components of the theory of planned behaviour, the socio-ecological model^(27)^, using deductive coding and other salient codes related to COVID-19, online grocery shopping and SNAP use to generate inductive coding. Two researchers independently applied the codebook (generating new codes as necessary) onto multiple transcripts until an inter-coder reliability of above 80 % was reached, after which the remaining transcripts were independently coded. MAXQDA was used for qualitative data analysis^(28)^.

The study’s protocol was submitted to the University of Maryland, Baltimore (UMB) Institutional Review Board (IRB), and it was deemed exempt for review under 45 CFR 46.101(b) (HM-HP-00090624). All participants provided written consent prior to completing the survey and written and verbal consent before participating in qualitative interviews or focus groups.

## Results

Table 1 provides descriptive characteristics of the sample by online grocery shopping uptake. Approximately half of respondents completed the survey in November or December 2020 (*n* 152), while the remainder completed the survey between January and March 2021 (*n* 158). Most households reported receiving SNAP benefits within the previous year (82 %). The majority of SNAP-participating households began receiving SNAP benefits prior to the COVID-19 pandemic, although almost one-fifth of SNAP households started receiving SNAP benefits during the pandemic.

**Table 1.** Demographics and Supplemental Nutrition Assistance Program (SNAP) participation among adopters and non-adopters of online grocery services in Maryland

	Never shopped online (*n* 133)		Late adopters (shopped online after the OPP) (*n* 97)		Early adopters (shopped online before the OPP) (*n* 80)		
Demographics	*n*	%	*n*	%	*n*	%	*P* value**
Age (years)							0·003
18–29	41	30·8	20	20·6	5	6·2	
30–39	60	45·1	47	48·5	50	62·5	
40–49	23	17·3	25	25·8	19	23·8	
50 or older	9	6·8	5	5·2	6	7·5	
Sex (female)*	125	94·0	91	93·8	71	89·9	0·483
Race							0·101
African American or Black	47	35·3	32	33·0	15	18·8	
White or Caucasian	70	52·6	58	59·8	55	68·8	
Other†	12	9·0	3	3·1	6	7·5	
Multiracial‡	4	3·0	4	4·1	4	5·0	
Locale§							0·995
Urban	115	86	84	86	69	86	
Suburban	7	5	6	6	5	6	
Rural	11	8	7	7	6	7	
In-store shopping frequency							0·001
≥ Once a month	30	22·6	14	14·4	7	8·8	
Every 2 weeks	35	26·3	18	18·6	12	15·0	
Once a week	68	51·1	65	67·0	61	76·2	
Intentions to shop online							
More in next 6 months	53	40·0	81	83·5	54	67·5	< 0·001
Will continue shopping online after the pandemic||	–	–	73	76·8	66	82·5	0·356

	Mean	sd	Mean	sd	Mean	sd	
Household size	4·41	1·51	4·10	1·36	4·53	1·55	0·088††
SNAP participation							
SNAP past 12 months	107	80·5	80	82·5	66	82·5	0·901
Started before COVID-19¶	89	83·2	61	77·2	57	87·7	0·250
Started after COVID-19	10	10·1	13	17·6	6	9·5	0·247
Other programmes:							
P-EBT	49	36·8	31	32·0	22	27·5	0·362
WIC	26	19·5	15	15·5	7	8·8	0·108
FRPSM	30	22·6	20	20·6	15	18·8	0·800

OPP, Online Purchasing Pilot; P-EBT, Pandemic Electronic Benefit Transfer; WIC, Special Supplemental Nutrition Program for Women, Infants, and Children; FRPSM, free and reduced-price school meal.

–, not applicable.

* One participant preferred not to say (*n* 309).

† Other race includes Asian or Asian American (*n* 7), Hispanic or Latino (*n* 9), Middle Eastern (*n* 1), Native American (*n* 0), Native Hawaiian (*n* 0) and other (*n* 3).

‡ Multiracial includes all those who selected more than one race/ethnicity.

§ Locale defined according to the 2010 Rural-Urban Commuting Area (RUCA) Codes: urban (metropolitan area core or high commuting), suburban (metropolitan area low commuting) and rural (micropolitan, small town or rural area).

|| Only asked to participants who reported previously shopping online (*n* 177, two missing responses).

¶ Pre-COVID-19 defined as between January and March 2020.

*P* value reported from **χ ^2^ test for independence comparing two or more categories, or ††ANOVA for difference in three means.

### Online grocery shopping experience

Most respondents (57 %) had prior online grocery shopping experience. More than half of those who reported shopping online started in the 6 months prior to the survey (i.e. after the onset of the pandemic and the start of the OPP), hereafter ‘late adopters’ (*n* 97), while the remainder (*n* 80) began shopping online more than 6 months prior to the survey, potentially before SNAP OPP (hereafter ‘early adopters’). Relative to early and late adopters of online shopping, participants who had never shopped online were younger and reported lower food shopping frequency.

The COVID-19 pandemic was cited as a situational trigger for starting online shopping use for many families:

*‘I started online [grocery] shopping because my household came down with COVID. We were very sick, and we weren’t able to go grocery shopping. When we were better, I still had heart issues and problems walking from COVID. So, ordering my groceries was — quite convenient. I can go online and pick out what I need, and it got delivered to my house. So that was the main reason I started shopping online’.*

Others mentioned the SNAP OPP as both a key factor in their decision to shop online and a determinant of where they shop online:

*‘Before this pandemic… before they started opening up more places where you could use the [SNAP] EBT, I wouldn’t have shopped at [retailer name] online for groceries, just for like basic household items’.*

### Purchasing behaviours and intentions associated with online grocery shopping

Table 2 shows the results of the robust Poisson regression models of key purchasing behaviours on uptake and timing of online grocery service use. Primary shoppers who reported shopping once per month or less were 34 % less likely to have ever shopped online than those who shopped online weekly (rate ratio (RR): 0·66, 95 % CI 0·46, 0·94), after controlling for covariates. However, grocery shopping frequency was not a predictor for early adoption of online grocery shopping compared with late adoption. Those who planned to shop online more in the near future were twice as likely to have ever shopped online (IRR: 2·10, 95 % CI 1·61, 2·74), but 37 % were less likely to be early adopters (RR: 0·63, 95 % CI 0·45, 0·89) compared with those not planning to shop more online after controlling for age, sex, household size, urbanicity and SNAP participation. No differences in intention to continue shopping online after the pandemic were observed between early and late adopters.

**Table 2.** Purchasing behaviours and intentions associated with online grocery shopping using Possion regression models

	Ever shopped online *v*. never shopped online (*n* 309)^a^		Early *v.* late adopters of online shopping (*n* 177)^b^	
Purchasing behaviour	RR	95 % CI	RR	95 % CI
Grocery shopping frequency†				
*Weekly	Ref	–	–	
*Twice per month	0·76	0·57, 1·02	1·05	0·65, 1·69
*About once per month	0·66*	0·46, 0·94	0·81	0·40, 1·67
Plan to shop online more in the next 6 months				
*No	Ref.	–	Ref	–
*Yes	2·10***	1·61, 2·74	0·63**	0·45, 0·89
Intention to continue shopping online after the pandemic				
*No	–	–	Ref	–
*Yes	–	–	1·20	0·76, 1·89

RR, rate ratio.

Results from separate multivariate robust Poisson regression models, controlling for age, sex, race, household size, urbanicity and food assistance programme participation.

a Missing data from one participant.

b Only asked to participants who reported previously shopping online (*n* 177, 2 missing responses).

*P < 0·05; **P < 0·01; ***P < 0·001.

† Grocery shopping frequency included all modalities (i.e. both online and in-store).

### Changes in attitudes towards in-store grocery shopping during the COVID-19 pandemic

**Figure 1.**
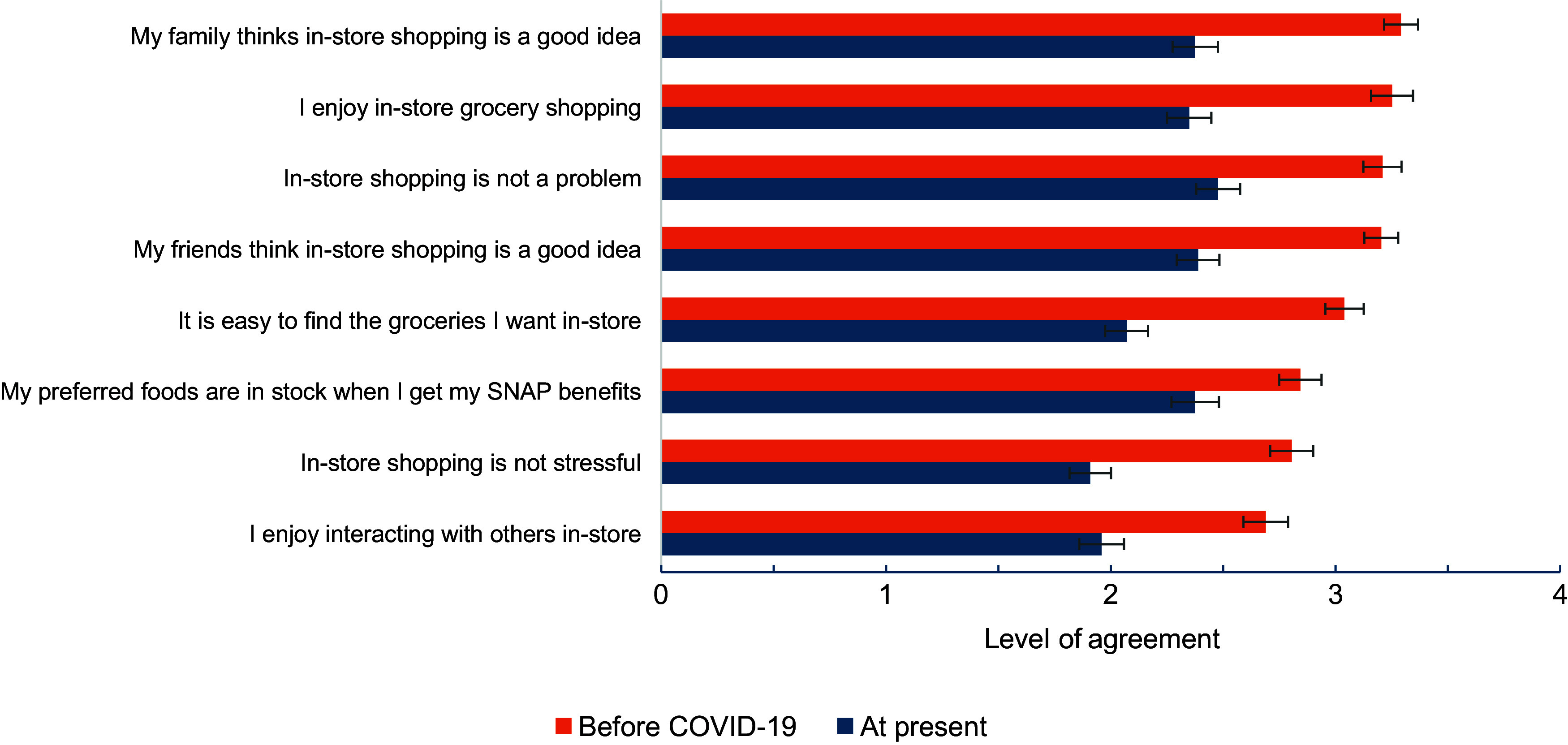
Attitudes towards in-store grocery shopping before and during the COVID-19 pandemic. Notes: Agreement with each statement was assessed using a Likert-based scale, in which ‘Strongly Disagree’ was coded as 1 and ‘Strongly Agree’ was coded as 4. Each bar represents the mean (95 % CI) level of agreement with the statement prior to COVID-19 and at the time of the survey. The level of agreement between each statement pre-COVID-19 and at the time of the survey were statistically significantly different at the *P* < 0·001 level for all statements, based on separate Wilcoxon signed rank tests. SNAP, Supplemental Nutrition Assistance Program.

Participants’ attitudes towards in-store grocery shopping dropped nearly a full point from self-reported pre-COVID-19 levels to the present across a variety of measures, including enjoyment of in-store grocery shopping, and families’ belief that in-store shopping is a good idea (Fig. 1). A one-point decrease is equivalent to a change in the four-point Likert scale from ‘Strongly Agree’ to ‘Agree’ or ‘Agree’ to ‘Disagree’. The largest mean decrease in score was for ease of finding groceries in-store (mean change (sd) = –0·97 (sd 1·04)), followed by families’ belief that in-store shopping is a good idea (mean change = –0·92 (sd 0·97)) (see online supplementary material, Supplemental Table 2). Two factors had mean scores indicating more disagreement than agreement during the pandemic, specifically ‘In-store grocery shopping is not stressful’ (mean: 1·91 (sd 0·81)) and ‘I enjoy going to the store to interact with other people’ (mean: 1·96 (sd 0·87)).

Qualitative findings supported quantitative results, as some participants reported enjoying the opportunities for social interaction provided by in-store grocery shopping and missing that aspect of the grocery shopping experience during COVID-19.

*‘I miss being able to get out with my mom, and we’re in the store and she’s getting her things, I’m getting my things, and we’re just having conversations, entertaining each other. I miss the actual human interaction… But I don’t want to get sick… I miss being around people, but I know I can’t be around people and still be healthy right now… So far, I’m good and healthy… So, I’ll deal with not being around people right now because me being a single parent, if something happens to me, who’s gonna take care of my kids?’*

Others had less favourable views of in-store shopping because of crowding and other stressors but noted that these concerns were heightened because of the pandemic.

*‘In-store grocery shopping is always kind of a struggle because it’s kind of crowded, and especially right now with the pandemic, that’s always a concern going into the grocery stores now’.*

Participants described the challenges they faced in finding the foods they needed, particularly during the early days of the pandemic. Some participants reported turning to online grocery retailers to purchase their groceries, while others found online grocery stores had similar shortages.

*‘With COVID, my stores were out of so much that I didn’t honestly know how we were going to make it, because I had to buy things that were triple the price, just so we would have something to eat v. being hungry. And it wound up being a lot of microwave stuff and a lot of not actual food that didn’t last as long but was triple the price. And every time somebody says “Oh, the COVID numbers are rising,” we get the same experience. So, the stores are empty, the online stores are empty, and there’s not really any options, especially living in a small area’.*

Participants frequently brought up family members’ feelings towards in-store shopping and perceptions of safety. Participants described older relatives’ concerns about in-store shopping during the pandemic and some mentioned assisting elderly parents and grandparents by shopping for them in-store or online.

*‘Like my mother, she’s afraid to go into stores… she’ll have double mask on, gloves, face shield, everything, before she walks into a store. So, I’ll say 98 % of her shopping is done online. Because going into a store she feels is unsafe. Then my grandmother, she’s 94; she hasn’t stepped in the store since this whole COVID mess started…’*

Families with young children discussed the hardships of buying groceries for the household on a budget and making food last for the whole month, especially when children were at home due to school closures. The additional SNAP benefit loaded to EBT cards for families with children attending schools that served free/reduced price meals (i.e. Pandemic Electronic Benefit Transfer (P-EBT)) alleviated, at least in part, concerns around making ends meet.

*‘That’s the one thing I can say has really made a big difference with this pandemic, the extra food stamps [SNAP] that I’ve got for my children, so thank you, government for thinking about us parents who have multiple children in the household who are eating up the place. I was like, “Man, I don’t know how I’m gonna afford it,” because I know when it’s summertime [and] they’re home more often than in the school year, they’re eating up everything, cereal’s gone in two days, milk’s gone in a day. I can’t keep up’.*

### Attitudinal and behavioural factors motivating continuation of online grocery purchase

Among participants who had shopped online, most (79 %) reported the intent to continue online shopping after the COVID-19 pandemic (Table 1). In a multiple Poisson regression model including the five attitudinal and behavioural factors derived from the confirmatory factor analysis, high scores on Factor 1 (Facilitators) were the strongest predictor of intent to continue online grocery shopping after the pandemic (RR: 1·54, 95 % CI 1·30, 1·84) and shop for groceries online more in the next 6 months than at present (RR: 1·54, 95 % CI 1·30, 1·83), after adjustment for demographic characteristics (Table 3). Participants with online grocery shopping experience who had higher scores for Fees (i.e. did not mind online shopping fees) were more likely to report planning to continue online grocery shopping after the pandemic (RR: 1·17, 95 % CI 1·04, 1·31). However, higher scores for Perceived Control, a measure of ease and comfort navigating online grocery platforms, were negatively associated with intent to increase the use of online grocery shopping services in the next 6 months in the full sample.

**Table 3.** Psychosocial predictors of intentions to online shopping after the pandemic

	Continue shopping online after COVID-19 (*n* 171)†		Shop online more in next 6 months (*n* 296)†	
	RR	95 % CI	RR	95 % CI
Factor 1: Facilitators	1·54***	1·30, 1·84	1·54***	1·30, 1·83
Factor 2: Fees	1·17*	1·04, 1·31	1·03	0·90, 1·18
Factor 3: Perceived control	1·04	0·88, 1·23	0·83*	0·70, 0·98
Factor 4: Access	1·03	0·88, 1·22	1·09	0·91, 1·30
Factor 5: Pick-up	0·89	0·78, 1·02	1·04	0·90, 1·22

RR, rate ratio; SNAP, Supplemental Nutrition Assistance Program; WIC, Special Supplemental Nutrition Program for Women, Infants, and Children; P-EBT, Pandemic Electronic Benefit Transfer.

Estimates from two multiple Poisson regression models with robust standard errors, adjusted for age, sex, race, household size, shopping frequency, urbanicity and nutrition assistance participation (SNAP, WIC and P-EBT). The model estimating the RR of shopping online more in the next 6 months also included prior experience shopping online.

Factors definitions: Facilitators (seven items, e.g. ‘Buying groceries online is helpful to me’), Fees (two items, e.g. ‘I don’t mind paying for service fees’), Perceived Control (nine items, e.g. ‘It does not take too long to search for specific products or labels online’), Access (two items, e.g. ‘I have access to a reliable internet connection to purchase groceries online’) and Pickup (two items, e.g. ‘Picking-up my groceries in store is more convenient than shopping in-store’).

*P < 0·05; ***P < 0·001.

† Continue shopping online after COVID-19 and shop online more in the next 6 months were variables generated from a four-point Likert scale question in which ‘Strongly Agree’ and ‘Agree’ were categorised as 1 and ‘Strongly Disagree’ and ‘Disagree’ as 0 (reference). Missing data were due to missing a response for a variable that composed one of the factors, *n* 4 and *n* 14, respectively.

In the qualitative sample, opinions about future online shopping use varied. Some individuals voiced interest in continuing to shop online after the pandemic, particularly for staples and packaged foods that are unlikely to spoil and not necessarily for groceries and fresh items.

*‘I think I’ll feel exactly the same way as I do now [after the pandemic]. I don’t think the pandemic is going to change how I want to pick my own food up, but I still do the toiletry items and like I said, bakery items and stuff like that. I could do those online and that will be fine’.*

Others preferred to return to in-store shopping, in part due to the perceived lack of control over food selection and substitutions when using online grocery services.

*‘Online shopping—I hope that’s not going to be “a thing,” because I’d rather just go into the market to get what I want, because they never give it to you right. You ask for gallon of milk and they bring you a quart and you’re like, ‘Wait a minute, what is this?’’*

## Discussion

This mixed-methods study adds to the growing literature on individual factors related to the adoption and continuation of online grocery shopping in the context of COVID-19 pandemic. In this sample of SNAP-eligible households with children, older primary shoppers (age ≥ 30) were more likely to be early adopters of online grocery shopping, corroborating trends reported in a national survey among individuals receiving SNAP^(15)^. The association between age and online grocery shopping use is mixed in the literature. Most of the studies conducted in the USA with low-income populations reported that older adults are less likely to shop online^(11,29)^. However, several recent studies have found that older adults were more likely to have adopted online grocery shopping after the onset of the pandemic^(5,30)^. One possible explanation is that older adults were at higher risk for COVID-19 and may have been more likely to avoid indoor and crowded spaces than younger adults.

The high proportion of participants reporting online grocery shopping for the first time during the pandemic corroborates the hypothesis that the pandemic acted as a catalyst for online grocery shopping. Indeed, pandemic-related health concerns (e.g. exposure to COVID-19 when shopping in-store) have been observed as contributors to online grocery shopping uptake in other studies^(6,31)^. However, while past research has evaluated the availability of online grocery services^(32)^ and communication efforts related to the SNAP OPP^(33)^, this study provides some of the first evidence to suggest the programme also contributed to increased uptake of online grocery shopping among low-income households. Specifically, while the pandemic-related concerns may have been a situational driver for online grocery shopping, the ability to use SNAP benefits online was an added facilitator which further drove online shopping towards specific large retailers. Additional research on awareness of the SNAP OPP and online grocery shopping use during the pandemic may help further understand the impact of the programme on increasing equitable access to healthy food via online grocery services.

We found that shopping frequency was positively associated with online grocery shopping adoption. This aligns with a prior study in Davis, California, which found that in-store shopping frequency was correlated with online grocery shopping, suggesting that the two modes of shopping are complementary^(34)^. It is possible that shoppers who buy groceries online may do supplemental grocery trips to local stores to purchase fresh foods, specialty items or products that are out of stock online. Although participants had to report purchasing > 50 % of their household’s groceries to be eligible for the present study, less frequent shoppers may share the responsibility for buying groceries with other household members and may have less motivation to shop online, as shopping in-store once or twice a month may not be perceived as a major inconvenience.

Interestingly, while intention to shop online more in the next 6 months was positively associated with prior online grocery shopping experience, there was a clear difference between early and late adopters of online grocery shopping. Early adopters of online grocery shopping were less likely to say that they intend to shop online more in the next 6 months than late adopters. Several possible explanations exist; early adopters may have tried online shopping in the first half of 2020, before the SNAP OPP, and had negative experiences, as participants in the qualitative study noted that they had experienced or heard about stock-outs and long wait times for delivery during the early stages of the pandemic. Alternatively, early adopters may already shop online often and have no perceived need to increase the frequency of their online grocery use.

During the pandemic, families reported more negative attitudes towards in-store grocery shopping compared with pre-pandemic, potentially influencing their decision to grocery shop online. Consumers have continued to report concerns tied to in-store grocery shopping related to the fear of infection from the COVID-19 virus, leading to a decrease in-store patronage^(35,36)^. While this risk perception led to an increase in stockpiling behaviours^(37)^ and decreases in shopping frequency, food-insecure households may not have had the financial resources to limit their in-store shopping by increasing food expenditures per shopping trip^(38)^. While our findings and prior studies suggest that most households with online shopping experience will continue shopping online^(3,5)^, some qualitative participants were unenthusiastic about online grocery shopping in the future. Future research to understand whether negative attitudes about in-store shopping and high use of online groceries services persist after the pandemic is resolved is needed.

Our findings indicate that participants who intended to continue or increase online shopping after the pandemic had greater perceptions of facilitator factors of online grocery shopping and were less concerned about fees. This finding corroborates a recent study among a nationally representative sample of the USA in which most respondents who had shopped for groceries online early in the pandemic planned to shop for groceries online in the future^(3)^. Our findings shed light on potential drivers that motivate individuals to grocery shop online, as individuals who have experienced shopping online may increase their self-efficacy and balance benefits (facilitators) against potential risks (fees) during their procurement decision. Nevertheless, service and delivery fees were still seen as a significant barrier to online grocery shopping among individuals of low income in this and other studies^(12,39,40)^. Future programmes should consider strategies to address barriers to food access imposed by online fees. Even in communities where grocery stores are physically available, equal access to all types of grocery shopping modes for families with low income is needed, so they can decide and use what is best for them in every given context. Additionally, our study also found that individuals with greater behvaioural control had lower intention to increase the use of online grocery shopping in the next 6 months. Although this may contradict the theory of planned behavior, it is possible that consumers with high control may be already purchasing more groceries online than instore and increasing the frequency is viewed as unnecessary.

The strengths of the current study are the mixed-method and exploratory design, which provided in-depth insight into online grocery shopping behaviours in a low-income sample. Additionally, this study was conducted in the context of the COVID-19 pandemic, which both led to the expansion of the SNAP OPP, and along with the OPP, acted as a situational factor influencing online grocery shopping uptake. The quantitative survey and the qualitative semi-structured interviews were informed by the theory of planned behavior to explain adoption of online grocery shopping.

However, this study also has some limitations. First, the sample consisted of low-income families eligible for SNAP with young children, so attitudes, barriers and intentions towards online grocery shopping for other populations (i.e. older adults) may be different and should be further investigated. Second, we only included English-speaking participants in our study. Third, we relied upon online recruitment strategies to enrol participants, which likely biased our sample towards individuals with Internet access and higher comfort with online technology. Fourth, this cross-sectional study simultaneously assessed attitudes towards in-store grocery shopping before and during the COVID-19 pandemic. However, individuals’ perceptions of their pre-pandemic attitudes may have been influenced by their recent experiences and memories, potentially introducing recall bias. Finally, to assess the relation between attitudinal and social determinants of online grocery shopping behaviours, we used the factor scores obtained in a confirmatory factor analysis (i.e. the latent structures of a set of observed variables) as observed values in the regression models, which likely results in underestimating the true standard errors of the estimates. However, confirmatory factor analysis was used as a data reduction technique widely used to test relations between observed variables and their underlying latent constructs.

### Conclusions

Although most participants who shopped online started after the onset of the COVID-19 pandemic, a high proportion intended to continue shopping online after the pandemic. Our findings suggest that online grocery services will remain an important source of food for SNAP-eligible households, affirming the need for policies that promote equitable access to healthy food online.

## Supporting information

Trude et al. supplementary material Trude et al. supplementary material

## Supplementary material

To view supplementary material for this article, please visit https://doi.org/10.1017/S1368980024002672
