# Supplementary material for: Changes in grocery shopping behaviour among low-income households during the COVID-19 pandemic
Source: Public Health Nutr. 2025 Jan 7;28(1):e26. doi: 10.1017/S1368980024002672 (PMC11822639; doi:10.1017/S1368980024002672)
Supplement: Trude et al. supplementary material [file S1368980024002672sup001.docx]

**Supplementary Material**

**Supplemental Table 1**. Purchasing Behaviors and Intentions Associated with Online Grocery Shopping using Logistic Regression Models

|  | Ever Shopped Online vs. Never Shopped Online (N=309)^a^ | |  | Early v. Late Adopters of Online Shopping (N=176)^b^ | |
| --- | --- | --- | --- | --- | --- |
| **Demographics** | OR | 95% CI |  | OR | 95% CI |
| Grocery Shopping Frequency^1^ |  |  |  |  |  |
| Weekly | Ref | - |  | - |  |
| Twice per Month | 0.51^*^ | 0.27, 0.96 |  | 1.16 | 0.45, 3.04 |
| About Once per Month | 0.39^*^ | 0.19, 0.80 |  | 0.71 | 0.22, 2.33 |
| Plan to Shop Online More in the Next Six Months |  |  |  |  |  |
| No | Ref. | - |  | Ref | - |
| Yes | 5.78*** | 3.36, 9.95 |  | 0.31** | 0.13, 0.75 |
| Intention to Continue Shopping Online after the pandemic |  |  |  |  |  |
| No | - | - |  | Ref | - |
| Yes | - | - |  | 1.42 | 0.61, 3.30 |

Results from separate multivariate logistic regression models, controlling for age, gender, race, household size, urbanicity, food assistance program participation.

Abbreviations: odds ration (OR), confidence interval (CI)

^*^p<0.05; ^**^p<0.01; ^***^p<0.001

^1^ Grocery shopping frequency included all modalities (i.e., both online and in-store).

**Supplemental Table 2**. T-test Statistics Comparing Means of In-store Grocery Shopping Attitudes, Social Norms, and Behaviors before and during the COVID-19 pandemic.

|  | Before COVID-19 | During COVID-19 | Change |  |  |  |
| --- | --- | --- | --- | --- | --- | --- |
| **Attitudes, Norms & Behaviors** | Mean (SD) | Mean (SD) | Mean (SD) | p-value | T test statistic | n |
| I enjoy in-store grocery shopping^a^ | 3.25 (0.82) | 2.35 (0.87) | -0.90 (0.95) | <0.001 | -16.34 | 298 |
| In-store grocery shopping is not stressful^b^ | 2.81 (0.84) | 1.91 (0.81) | -0.90 (1.01) | <0.001 | -15.42 | 297 |
| In-store grocery shopping is not a problem for me^b^ | 3.21 (0.75) | 2.48 (0.86) | -0.73  (1.01) | <0.001 | -11.85 | 297 |
| My family thinks that buying groceries in the store is a good idea^a^ | 3.29 (0.67) | 2.38 (0.89) | -0.92 (0.97) | <0.001 | -16.15 | 298 |
| My friends think that buying groceries in the store is a good idea^c^ | 3.20 (0.66) | 2.39 (0.83) | -0.82 (1.02) | <0.001 | -13.73 | 295 |
| It is easy to find all the groceries I want in stores^b^ | 3.04 (0.75) | 2.07 (0.84) | -0.97 (1.04) | <0.001 | -16.05 | 297 |
| I enjoy going to the store to interact with other people^b^ | 2.69 (0.87) | 1.96 (0.87) | -0.73 (1.02) | <0.001 | -12.38 | 297 |
| When I receive my SNAP benefits, the foods that I usually buy are in stock^d^ | 2.84 (0.76) | 2.38 (0.85) | -0.47 (0.92) | <0.001 | -8.01 | 250 |

A one-point decrease is equivalent to a change in the 4-point Likert-scale from “Strongly Agree” to “Agree” or “Agree” to “Disagree.” P-value and T-test statistic calculated using paired t-tests.

**Supplemental Table 3**. Logistic Regression Models of Psychosocial Predictors of Intentions to Online Shopping after the Pandemic Intent to Continue Use Online Shopping after the Pandemic

|  | Continue Shopping Online After COVID (N=171)^a^ | | Shop Online More in Next 6 Months (N=296)^b^ | |
| --- | --- | --- | --- | --- |
|  | OR | 95% CI | OR | 95% CI |
| Factor 1: Facilitators | 70.11 | 13.73, 358.15 | 4.29 | 2.73, 6.73 |
| Factor 2: Fees | 6.68 | 3.15, 14.14 | 1.64 | 1.18, 2.28 |
| Factor 3: Perceived Control | 3.45 | 1.82, 6.56 | 0.99 | 0.72, 1.37 |
| Factor 4: Access | 15.09 | 4.85, 46.96 | 2.79 | 1.78, 4.36 |
| Factor 5: Pick-up | 3.21 | 1.66, 6.20 | 1.56 | 1.09, 2.23 |

Estimates from separate multiple logistic regression models, adjusted for age, sex, race, household size, shopping frequency, urbanicity, nutrition assistance participation (SNAP, WIC, P-EBT), and prior experience shopping online. The model estimating the OR of shopping online more in the next 6 months also included prior experience shopping online.

Abbreviations: odds ratio (OR), confidence interval (CI)

^a^ Continue shopping online after COVID and Shop online more in the next 6 months were varibales generated from a 4-point Likert scale question in which “Strongly Agree” and “Agree” were categorized as 1 and “Strongly Disagree” and “Disagree” as 0 (reference). Missing data were due to missing a response for a variable that composed one of the factors, n=4 and n=14, respectively.

Factors definitions: Facilitators (7 items, e.g.: “Buying groceries online is helpful to me”), Fees (2 items, e.g.: “I don’t mind paying for service fees”), Perceived Control (9 items, e.g.: “It does not take too long to search for specific products or labels online”), Access (2 items, e.g.: “I have access to a reliable internet connection to purchase groceries online”), and Pickup (2 items, e.g.: “Picking-up my groceries in store is more convenient than shopping in-store”).
